# Methods of determining optimal cut-point of diagnostic biomarkers with application of clinical data in ROC analysis: an update review

**DOI:** 10.1186/s12874-024-02198-2

**Published:** 2024-04-08

**Authors:** Mojtaba Hassanzad, Karimollah Hajian-Tilaki

**Affiliations:** 1 Student Research Center, Research Institute, Babol University of Medical Sciences, Babol, Iran; 2 Department of Biostatistics and Epidemiology, School of Public Health, Babol University of Medical Sciences, Babol, Iran; 3 Social Determinant Health Research Center, Research Institute, Babol University of Medical Sciences, Babol, Iran

**Keywords:** ROC analysis, Optimal cut-point, Youden index, Euclidean index, Product method, Index of union, Diagnostic odds ratio

## Abstract

**Introduction:**

An important application of ROC analysis is the determination of the optimal cut-point for biomarkers in diagnostic studies. This comprehensive review provides a framework of cut-point election for biomarkers in diagnostic medicine.

**Methods:**

Several methods were proposed for the selection of optional cut-points. The validity and precision of the proposed methods were discussed and the clinical application of the methods was illustrated with a practical example of clinical diagnostic data of C-reactive protein (CRP), erythrocyte sedimentation rate (ESR) and malondialdehyde (MDA) for prediction of inflammatory bowel disease (IBD) patients using the NCSS software.

**Results:**

Our results in the clinical data suggested that for CRP and MDA, the calculated cut-points of the Youden index, Euclidean index, Product and Union index methods were consistent in predicting IBD patients, while for ESR, only the Euclidean and Product methods yielded similar estimates. However, the diagnostic odds ratio (DOR) method provided more extreme values for the optimal cut-point for all biomarkers analyzed.

**Conclusion:**

Overall, the four methods including the Youden index, Euclidean index, Product, and IU can produce quite similar optimal cut-points for binormal pairs with the same variance. The cut-point determined with the Youden index may not agree with the other three methods in the case of skewed distributions while DOR does not produce valid informative cut-points. Therefore, more extensive Monte Carlo simulation studies are needed to investigate the conditions of test result distributions that may lead to inconsistent findings in clinical diagnostics.

## Introduction

**Fig. 1:**
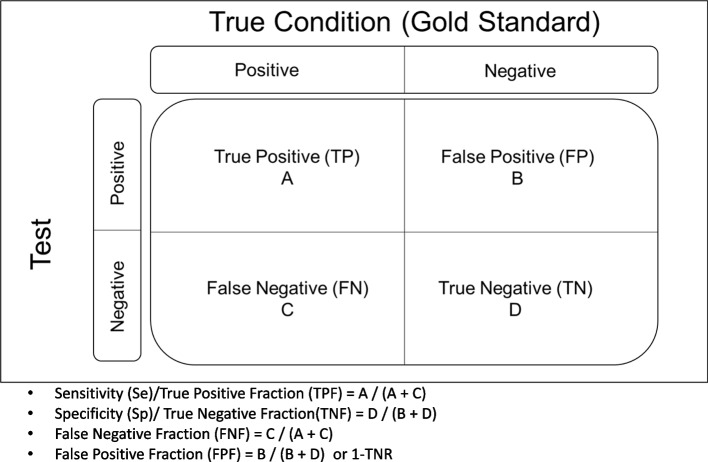
A summary of test result and its true condition

One of the most important medical challenges is the clinical evaluation of diagnostic tests, which is of interest to clinical experts and statistical researchers. The gold standard methods are likely to be invasive and costly. Therefore, an evaluation of new diagnostic tests is very important. If the result of the diagnostic test is binary, sensitivity (Se) and specificity (Sp) are used as measures of the diagnostic accuracy. Se (true positive rate) refers to the probability of a positive test result for the persons with Target Condition (TC). The Sp (true negative rate) is the probability that the test result is negative, provided the person is without TF [1–3]. From a clinical perspective, in addition to Se and Sp, two other measures, the positive and negative predictive values, are of interest to clinicians. The negative predictive value (NPV) indicates the probability that a person is without TC if the test result is negative. The positive predictive value (PPV) denotes the probability of having TC if the test result is positive. The PPV and NPV are clinically important but they are influenced by the prevalence of TC in target population. Clinicians are interested in the PPV and NPV and want to assess the likelihood that a person is with TC or without TC based on the test results [2, 3]. As a rule, the results of the gold standard status and the test are summarized in Fig. 1 as follows:

However, no longer diagnostic tests are confined to positive/negative results. Many biomarkers in laboratory tests yield results on a continuous scale. The Receiver Operator Characteristic (ROC) curve analysis is a method of choice to determine the diagnostic accuracy (area under the ROC curve-AUC) and partial area [3]. However, from clinical decision-making, it is interesting to define an optimal cut-point on continuous biomarkers. Several methods for optimal cut-point selection have been proposed [4–9]. The choice of priority between these methods is a matter of interest in clinical practice. Thus, the objective of this study was to provide an updated extensive review of ROC analysis and the methods of cut-point selection of biomarkers with application using clinical data. In the following sections, first, we provided an overview of ROC analysis for diagnostic biomarkers. In particular, we focused on the different methods of cut-point selection for laboratory diagnostic test. We illustrated the five popular methods of cut-point selection with clinical data. The consistency and inconsistency of findings were discussed depending on the distribution of test results in diseased and healthy populations.

### Overview of ROC curve for quantitative biomarker

Many diagnostic markers in modern medicine are quantitative. Various cut-off points can be considered for them, from which the Se and Sp for each of the points are derived [1]. The trade-off between (1-Sp) and (Se) should be plotted on a coordinate system, and the process of changes in Se versus (1-Sp) is called the receiver operating characteristics (ROC) analysis curve [2, 3]. This curve shows the diagnostic accuracy of the test and expresses clinically and statistically the area under the curve (AUC) of the diagnostic power of the test, which corresponds exactly to the Wilcoxon statistic [10]. Historically, this was used in radars during World War II to identify the point as a target or object (true positive or Se) amidst the clutter (FP or 1-Sp) on the ROC [11, 12]. It was later used by Lusted in radiology to characterize pulmonary tuberculosis and to determine the correlation of FP and FN findings in several studies on the interpretation of chest radiographs and more recently in clinical epidemiology to determine the diagnostic accuracy of biomarkers [13]. This graph therefore clearly determines the presence or absence of the desired result for objects or persons. In the medical and statistical literature, this ROC curve is often used to evaluate the diagnostic significance of quantitative markers. However, the most important thing about the ROC curve is that it can be used to determine the optimal cut-off point for quantitative biomarkers.

**Fig. 2:**
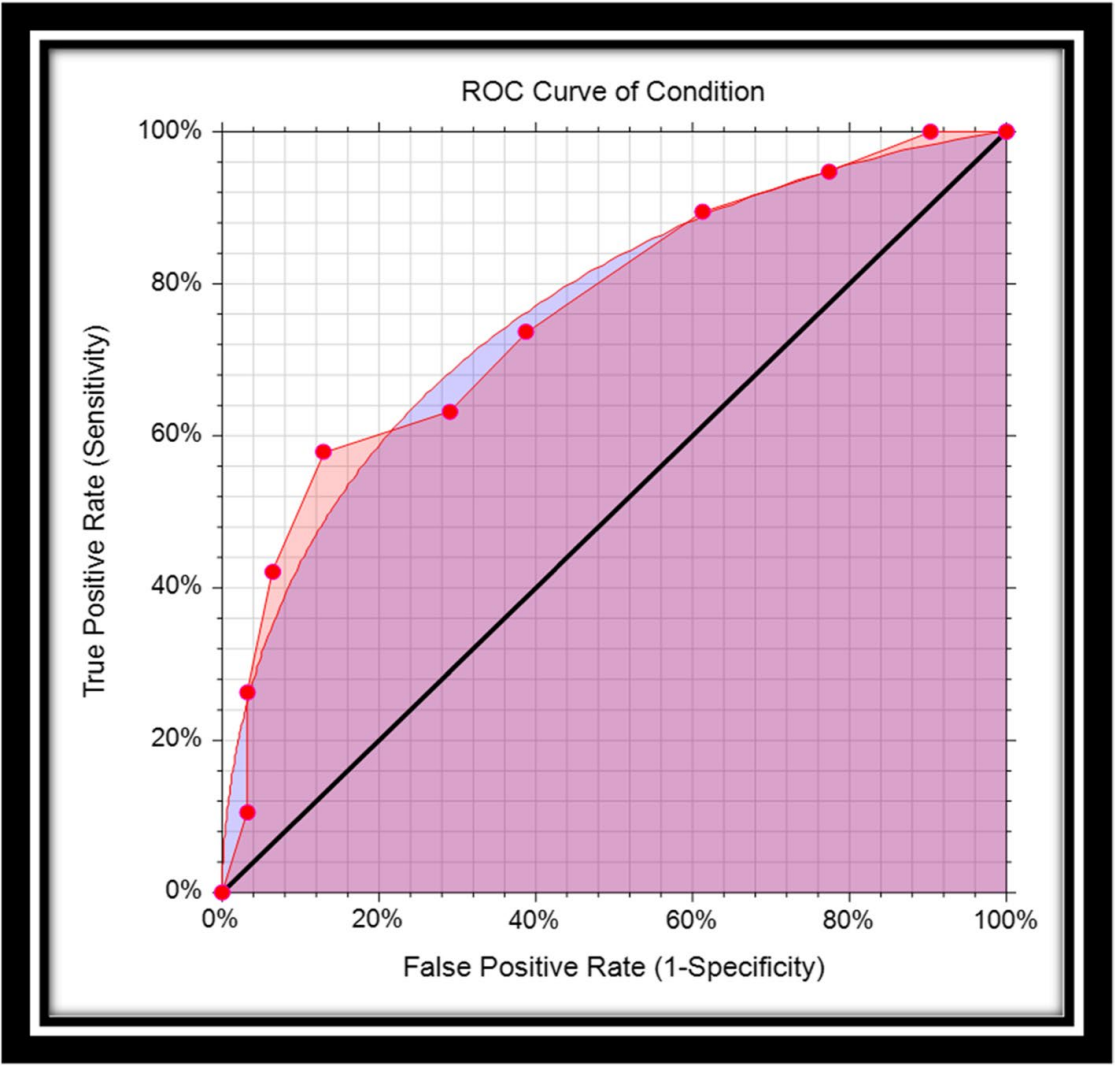
Empirical and smooth ROC curve

The structure of the ROC graph was shown in Fig. 2. The ROC graph is plotted in a 1 × 1 square, where the vertical axis corresponds to the Se rate, but the horizontal axis of this graph corresponds to the FP rate. Within this square, there is a curve and a diameter [3, 14]. The lower left corner is Se = 0 & Sp = 1, i.e. the highest possible cut-off value of the test. As we move from the lower left corner to the upper right corner, the Se increases but the Sp decreases. As a result, the cut-off value gets lower and lower, and at the end of the upper right corner of the square, the Se and Sp are 1 and 0, respectively, i.e. the lowest possible cut-off value for this test [11]. The stricter the criteria for determining a positive result, the more points on the curve shift downwards and to the left. If, on the other hand, a looser criterion is applied, the point on the curve shifts upwards and to the right [15].

### Interpretation of different shapes of ROC curve

If the ROC curve lies above the square diameter, this means that the test correctly determines the difference between the two target populations (healthy people, and sick people). The closer this curve is to the upper left corner, the better the diagnostic significance. Even if this curve is placed in the left-hand corner with the indication (0.1), the test has full diagnostic significance (Se = Sp = 1) [11, 16]. If the curve is placed on the diameter, this means that the two identified populations have been randomly classified [11, 16]. If the curve is below the diameter, this means that the test results are completely misleading. So the basic idea of this graph is that all points should be near the upper left corner. However, among all these points, we should look for the point with the best cut-off value, as this point is used to determine the threshold value for distinguishing between two healthy and diseased populations.

### Area Under the Curve (AUC)

The area under the ROC curve is abbreviated as AUC. The AUC can be calculated either parametrically under binormal distributions (or other pairs of distributions of test results) [17–19] or nonparametrically (i.e. empirically, without making any distributional assumptions of test results) [18–20]. Several methods have been suggested to calculate the standard error of AUC either parametrically or nonparametrically. The other index is the partial area that might be interested at clinical relevant range of false positive [19–22]. The AUC is one of the indicators of diagnostic accuracy when comparing diagnostic tests in the ROC analysis. The AUC summarizes the entire position of the ROC curve and is not dependent on a specific operating point [3]. AUC is interpreted in the following two ways: The statistical concept of AUC is the probability that the criterion value of an individual randomly drawn from a population of individuals with a diseased condition is greater than the criterion value of another individual randomly drawn from a population of individuals with a healthy condition [18], or that it is interpreted as the mean true positive rate (average Se) over all possible FP rates. One of the purposes of the ROC curve is to compare two or more diagnostic tests in the ROC analysis. Of course, the higher the AUC value, the higher the accuracy of the test. The maximum value that the AUC can have is 1, which means that the diagnostic test correctly and completely distinguishes two populations (this is the case when the distribution of the test result for two populations, namely healthy and diseased, does not overlap at all). If the AUC is 0.5, this means that the differentiation is random and the ROC curve lies exactly on the square diameter.

### Parametric and nonparametric AUC

The most popular parametric model is the binormal model that assumes the distributions of test results in a healthy and sick population follow a Gaussian distribution with different means and standard deviation. Based on this assumption, a smooth ROC curve can be driven, and the AUC can be calculated with a closed formula as follows:

$$AUC=\phi (\frac{\mu 1-\mu 0}{\sqrt{{ \sigma 1}^{2}+{\sigma 0}^{2}}})$$

Where, µ1, µ0 the mean of the diseased and healthy population and σ1, σ0 are the standard deviation of the diseased and healthy population respectively, and ϕ is, the cumulative standard normal distribution function.

The nice property of the ROC curve is that AUC is invariant to any monotonic transformation of the decision scale. However, binormal model is a theoretical model, and it is not observed in real life, in particular when the sample size is small. The alternative nonparametric approach is more practical for non-binormal data. The nonparametric Wilcoxon statistics provide an estimate of the trapezoidal role of AUC. Hajian-Tilaki and Hanley showed practical calculation of non-parametric AUC based on the pseudo-accuracy and its sampling variability [10]. This latter approach is more convenient for non-Gaussian data with a small sample size. For example, Fig. 2 provides binormal AUC (smooth cure) and nonparametric AUC (empirical AUC). However, as we pointed out already, the AUC is the Se averaged over all possible cutoffs and thus the comparison of two diagnostic tests based on AUC can be misleading when they are crossing each other and results in a wrong conclusion because the AUC is the sensitivity averaged over all possible cutoffs. In this situation, the Se at a given relevant range of FPF and at an optimal cut-point is interesting.

### Main issues of performing diagnostic test

The main issues of diagnostic tests are how the test results will be used in real life (is the test for “rule in” or “rule out”, what is the target population? What are the next steps given the positive test results, and so on). Although the Youden index provides beautiful statistical properties and clinical interpretations, it may not be recommended in real life for cut-off selection because it assumes an equal weight for Se and Sp. For example, in a screening test for cancer, the false negative results are much more serious than false positive because the positive results usually should be confirmed by other tests and procedures. Medical diagnostic tests can have different indications for use as a diagnosis, prognosis, monitoring, risk assessment, treatment choice and so on. For example, for the “rule-out” test for cancer, a typical cutoff is a prespecified level of Se (for example, 99%) and a clinical acceptable level of Sp. Another issue of applying a diagnostic test for evaluation of Se and Sp is that a test should be applied from the same source to the target population. For example, for diagnosing Alzheimer's Disease (AD), the target population might be subjects with memory problems and with and without AD. If one calculates the Sp based on the “healthy” subjects, it provides a very biased estimation. We should emphasize that the best methods of cut-point selection with desirable statistical properties and clinically relevant, cannot solve the problems of design in performing diagnostic test. Another bias may arise with further work-up, when primarily the test result is negative. The results of a diagnostic test affect the gold standard test (or reference test) that is used to verify the test results. This type of bias sometimes called “verification bias” or “work-up bias”. The partial verification may occur when only those with a positive test receive the reference standard test and differential verification occurs where a different reference test is used depending on whether the preliminary test was positive or negative. Blinding work-up may reduce such bias.

### Rationale of optimal cut-off value for quantitative diagnostic biomarkers

When a quantitative diagnostic test is performed, two groups cannot be completely distinguished due to the overlap of test results in the group of patients and healthy individuals [23]. An example: Imagine two hypothetical distributions that refer to a situation in which the average test result is 80 in the patient group and 60 in the non-patient group. If the cut-off value is set to 70 in this situation, people with the disease whose test result is below 70 are incorrectly classified as not having the disease (FN). However, if the doctor lowers the cut-off value to 65 in order to increase the Se of the test, the number of people who test positive increases (the Se increases), but the number of FP results also increases. In general, it is important to determine a cut-off value with adequate Se and Sp, as the use of less stringent criteria to increase Se leads to a trade-off in which Sp decreases.

### Methods of determining optimal cut-off value

One of the most important applications of the ROC curve is the determination of the optimal cut-off value for quantitative biomarkers. The search for the optimal cut-off value is not only about maximizing Se and Sp but also about finding a suitable compromise between the two based on various criteria [11]. When a disease is highly contagious or associated with severe complications, Se is more important than Sp. In contrast, Sp is more important than Se when it comes to whether a test is expensive or risky. If there is no trade-off between Se and Sp, or if both are equally important, it makes the most sense to maximize both [11]. Several methods have been introduced to determine the optimal cut-off point, but some of them are very common and it should be noted that each of them has unique assumptions, and the selection of each one is based on the importance of the Se versus the Sp of the test. The most important of these methods are as follows:

1. Youden’s J statistic
2. Euclidean distance
3. Index of union (IU)
4. Cost approach
5. Positive likelihood ratio (LR +) and negative likelihood ratio (LR–)
6. Maximum product of sensitivity and specificity
7. Number needed to misdiagnose (NNM)
8. Analytical method
9. Diagnosis Odds Ratio
10. Min *P*-Value

### Youden’s J statistic

The Youden index uses the maximum vertical distance of the ROC curve from the point (X, Y) on the diagonal (random line). In fact, the Youden index maximizes the difference between the Se and FP rate, in other words, it maximizes the percentage of Net correct classification:

$$\text{Youden Index} = \text{Se} + \text{Sp} - 1 = \text{Se} - (1 - \text{Sp})$$

Therefore, the optimal cut-off point is calculated by maximizing Se + Sp at different cut-off points [15, 23].

### Euclidean distance

Another way to determine the optimal cut-off value is to use the Euclidean distance from the coordinates (0, 1) in the left corner of the ROC space. In this method, the optimal cut-off value is determined according to the basic principle that the AUC value should be maximum. Therefore, the distance between the coordinate (0, 1) and the ROC curve should be minimized. The Euclidean distance is defined as follows:

$$\sqrt{{\left(1-Se\right)}^{2}+{\left(1-Sp\right)}^{2}}$$

The point at which this value is minimized is considered the optimal cut-off value [3, 23].

### Index of Union (IU)

The Index of Union (IU) uses the absolute value difference between the diagnostic measure and the AUC value to minimize the misclassification rate, which is calculated using the following formula.

$$IU=\left|Se-AUC\right|+\left|Sp-AUC\right|$$

IU is a method to find the point at which Se and Sp are maximized simultaneously. This is similar to the Euclidean distance. The difference, however, is that it minimizes the absolute value differences between the AUC value and the diagnostic measurements (Se and Sp), and this index also minimizes the difference between Se and Sp. The cut-off point at which the IU is minimized is optimal. This method does not require complex calculations, as it only checks whether the Se and Sp at the optimal cut-off value are sufficiently close to the AUC values or not. Furthermore, in most cases, IU has a better diagnostic performance than other methods [5].

### Cost approach

The cost approach is a method for determining the optimal cut-off value that takes into account the benefits of correct classification or the costs of misclassification. This method can be used when the costs of true positive (TP), true negative (TN), FP, and FN in a diagnostic test are known [24]. There are two ways to determine the cut-off value using the cost approach: to calculate the cost itself or use the cost index (f_m_).

$$\begin{aligned}{\text{Cost}}=& {{\text{C}}}_{{\text{FN}}}\left(1-{\text{Se}}\right)\mathrm{ Pr}+{{\text{C}}}_{{\text{FP}}} \left(1-{\text{Sp}}\right)\left(1-{\text{Pr}}\right)+{{\text{C}}}_{{\text{TP}}}\ \mathrm{Se Pr}+ {{\text{C}}}_{{\text{TN}}}\ \mathrm{ Sp }\left(1-{\text{Pr}}\right)\\ {f}_{{\text{m}}}=&{\ \text{Se}}- \left(\frac{1-{\text{Pr}}}{{\text{Pr}}}\times \frac{{{\text{C}}}_{{\text{FP}}}-{{\text{C}}}_{{\text{TN}}}}{{{\text{C}}}_{{\text{FN}}}-{{\text{C}}}_{{\text{TP}}}}\right) \left(1-{\text{Sp}}\right)\end{aligned}$$

where Pr is the prevalence and C_TN_, C_FP_, C_TP_, and C_FN_ refer to the costs of TNs, FPs, TPs, and FNs, respectively. These four costs should be mentioned in a common unit. When the cost index (*f*_m_) is maximized, the average cost is minimized, and this point is regarded as the optimal cut-off value [24].

Another method to determine the optimal cut-off value in terms of costs is to use the misclassification cost term (MCT). Considering only the prevalence of the disease, C_FN,_ and C_FP_, the point at which the MCT is minimized is determined as the optimal cut-off value [6, 23].

$${\text{MCT}}=\frac{{{\text{C}}}_{{\text{FN}}}}{{{\text{C}}}_{{\text{FP}}}}\times \mathrm{Pr }\left(1-{\text{Se}}\right)+\left(1-{\text{Pr}}\right)\left(1-{\text{Sp}}\right)$$

### Positive likelihood ratio (LR +) and negative likelihood ratio (LR–)

Positive likelihood ratio ($L{R}^{+}$) is the ratio of true positives to FPs and negative likelihood ratio ($L{R}^{-}$) is the ratio of FNs to true negatives. Researchers can choose a cut-off value that either maximizes $L{R}^{+}$ or minimizes $L{R}^{-}$. The larger the $L{R}^{+}$ is, the more information it has for the diagnostic test, but with the $L{R}^{-}$ it is exactly the opposite: if it is close to zero, the test performs better [23, 24].

### Maximum product of sensitivity and specificity

In this method, the point at which the product of Se and Sp reaches the maximum is regarded as the optimal cut-off value [7, 15].

$$\mathrm{Product\,}=\mathrm{\,Max\,}(\mathrm{Se\,}\times\,\mathrm{\,Sp})$$

### Number needed to misdiagnose (NNM)

This method refers to the number of patients in whom a misdiagnosis is estimated when a diagnostic test is performed. In other words: If number needed to misdiagnose (NNM) = 10, this means that ten people would need to be tested to find one misdiagnosed patient. The higher the NNM (maximize), the better the test performance [11].

### Analytical method

This method is related to the NNM, with the difference that the NNM assumes that the costs of FP and FN are equal, but otherwise, there is a new formula where FN equals C equals FP, resulting in a weighted NNM. To find the most appropriate cut-off value, the weighted NNM can be maximized to account for both the proximity of test results to gold standard results and the cost of misdiagnosis (FP and FN) [11].

### Diagnostic odds ratio (DOR)

The diagnostic odds ratio (DOR) is calculated by dividing the $L{R}^{+}$ by the $L{R}^{-}$. By maximizing the LR + and minimizing the LR-, the optimal cut-off point can be determined. Note that the $L{R}^{+}$ is between 0 and + ∞, but the $L{R}^{-}$ is between 0 and 1. The DOR is between 0 and + ∞; if DOR = 1, it means that the DOR shows no relationship between the test results and the target conditions. But if both FP and FN are zero, the test has both Se and Sp of 100% [8, 15].

$$\begin{aligned} &DOR= \frac{{LR}^{+}}{{LR}^{-}}=\frac{\frac{Se}{1-Sp}}{\frac{1-Se}{Sp}}=\frac{Se\times Sp}{\left(1-Se\right)\left(1-Sp\right)}\\ & {\text{Log}}\left(DOR\right)={\text{Log}}\left(\frac{Se}{1-Se}\right)+Log\left(\frac{Sp}{1-Sp}\right)={\text{logit}}\left(Se\right)+{\text{logit}}(Sp) \\&SE\left({\text{Log}}\left(DOR\right)\right)=\sqrt{\frac{1}{TP}+\frac{1}{FN}+\frac{1}{FP}+\frac{1}{TN}} \end{aligned}$$

The log(DOR) has an approximately normal distribution and with SE(LOG(DOR)) you can obtain a confidence interval for LOG(DOR) and then calculate the limit value of the confidence interval for DOR by subtracting the antilogarithm. Obviously, the lack of FP and FN data at a given cut-off value can lead to low accuracy of LOG(DOR) estimation [8, 15]. The DOR has a disadvantage: it produces a very low or very high cut-off point. One of the limitations of the statistical behavior of DOR is that it is associated with a higher mean square error (MSE) in the right tail, resulting in an unstable measurement. Therefore, it is suggested to minimize the MSE instead of maximizing it. Hajian-Tilaki has presented a graphical method based on a study relying on the distribution of data over the population and shown that the DOR is not compatible with Youden and Euclid’s methods in determining the optimal cut-off point and is sometimes noninformative under certain conditions [15].

### Minimum *P*-value approach (min P)

In this method, all cut-off points resulting from the trade-off between Se and FP are determined, the *P*-value is calculated for each of them and the point with the smallest *P*-value is selected as the optimal cut-off point [5, 9]. Statistically, this *P*-value is driven from a chi-square distribution with one degree of freedom.

### A review of performance of different methods of optimal cut-point

To compare different methods for determining the optimal cut-off point, various population-based and Monte Carlo simulation studies were conducted, the results of which are summarized in Table 1. In the study by Hajian-Tilaki, the four methods were compared based on different distributions of data in patients and healthy individuals, including Youden's J-statistic, Euclidean distance, product of Se and Sp, and diagnostic odds ratio (DOR). Of these methods, only the DOR differed from the other methods. However, the cut-off point in other methods was almost similar and consistent under binormal distributions, but when using DOR, the cut-off point is too high or too low, which is not reliable. That is if the model was binormal with similar variances for two groups, the DOR metric curve was U-shaped, and maximizing it gives the optimal cut-off point on the extreme critical values. But when the variances were different, the DOR increased exponentially, so the optimal cut-off point was very high, but when the healthy group had more variance, the optimal cut-off point was very low; in the cases where the bilogistic model was considered to have equal variance, the DOR was fixed at different cut-off points, but in the case where the variance of the patient group was larger, it had a linear relationship (straight line) with a positive slope at different cut-off points, making the optimal cut-off point very high. As an advantage of ROC analysis for quantitative diagnostic tests, it is recommended to use the Youden index, the Euclidean index, or the product of Se and Sp to obtain optimal cut-off values [15].

**Table 1:** Summary of comparing different methods on determining optimal cut-off in population based and simulation studies

Author	Year of publication	Underlying distribution	Sample size		Performance
			D	ND	
Hajian-Tilak [15]	2018	Normal Logistic	✖	✖	DOR had a poor performance in cut-point selection
Unal [5]	2017	Normal Gamma	50 100 200	50 100 200	IU had the least MSE and relative bias compared with other methods
			50 50 50	100 150 200	
Habibzade et al. [11]	2016	✖	✖	✖	Considering the costs of FP and FN, the analytical methods had a better performance than others
Perkins et al. [26]	2006	✖	120	120	The difference between the Youden and Euclidean was negligible in determining optimal cut-point
Liu [7]	2012	Normal	50 100 150	50 100 150	Youden index had a higher MSE than Euclidean and product methods
			100 200 200	200 100 200	
Gerke et al. [27]	2022	Normal	✖	✖	✖
Rota et al. [9]	2014	Normal Gamma	50 100 200	50 100 200	Eucliden and product methods had lower MSE and relative bias than Youden index
			50 50 50	100 150 200	

*D* Diseased, *ND* Nondiseased, *DOR* Diagnostic Odds Ratio, *IU* Index of Union, *MSE* Mean Square Errors, *FP* False Positive, *FN* False Negative

In the Ünal simulation study, methods such as Youden's J-statistic, minimum *P*-value, maximum product of Se and Sp, Euclidean distance and IU were applied to the simulated data. By comparing MSE, relative bias, bootstrap SD, coverage and average length, it was found that IU and Euclidean distance determine the best cut-off point, but the author rather recommends IU due to its clinical significance and easier understanding for clinicians [5].

In the simulation study by Rota et al., the comparison and calculation of different methods for determining the optimal cut-off value was carried out in the form of a simulation, as in the Ünal study, with the difference that the IU method was not used. In the report on Euclidean distance, almost better performance in terms of MSE, bias, etc. was shown in estimating the optimal cut-off point, although the author did not declare this method as the best method for determining the optimal cut-off point [9].

Habibzadeh et al. used methods such as Se = Sp (this method determines the point corresponding to the optimal cut-off point resulting from the maximum product of Se by Sp), Bayesian approach, Youden's J-statistic, Euclidean distance, maximum weighted NNM, and an analytical method using Hooper et al.’s population-based distribution data [25]. They considered MCT and had information such as the cost of FP and FN and pretest probability, a more appropriate optimal cut-off point could be determined by maximum weighted NNM and analytical methods [11].

Perkins and Schisterman evaluated the Youden and Euclidean distance methods using population-based distribution data. Both methods reached almost the same optimal cut-off point, but in their study, the Youden method was recommended more due to its clinical concept, as it increases the rate of correct classification and decreases the rate of misclassification, although the Euclidean method has more geometric significance, less clinical significance and also maximizes the rate of misclassification [26].

Liu used simulation data with a normal distribution [7]. The Youden, Euclidian, and Product methods were used to determine the optimal cut-off point. The comparison criterion for these three methods was the MSE, which was lowest for the Product and Euclidian methods, while the Youden method had the highest MSE, especially when the classification accuracy was low [7].

Moreover, Gerke et al. utilized simulation data with four different scenarios, including the healthy and sick groups with two normal distributions with different mean and variance, the healthy group with normal distribution and the sick group with gamma distribution, and the last scenario in which the healthy group had an exponential distribution and the sick group had a gamma distribution. The Youden, Euclidean, and Product methods were used to calculate the true optimal cut-off value. The result was that these three methods had the same true optimal cut-off value only in the first scenario, in which the two groups were normally distributed but had different mean values (in the other scenarios, however, there was a difference of one hundredth) [27].

### Statistical software for ROC curve analysis

Statistical programs used to perform ROC curve analysis included various commercial software programs such as IBM SPSS, MedCalc, SAS, Stata, and NCSS as well as open-source software (OSS) such as R and Metz-ROC [23]. IBM SPSS, the most widely used commercial software, can perform basic statistical analysis for ROC curves, such as plotting ROC curves and calculating AUC and CI with statistical tests, but it lacks the comparison of two correlated ROC curves. This output-based software does not report the optimal cut-off point, but only gives the non-parametric ROC curve, AUC, 95% CI, and test (H0: AUC = 0.5, H1: AUC ≠ 0.5). Stata provides several functions for analyzing ROC curves, including partial AUC (pAUC) [28], comparing multiple ROC curves, determining the optimal cut-off value using the Youden index, and comparing two or more output AUCs. MedCalc provides a sample size estimate for a single diagnostic test and includes various analysis methods to determine the optimal cut-off value, but does not provide a function to calculate pAUC. In terms of NCSS, this software can: generate empirical and binormal ROC curves, calculate AUC, determine the cut-off value, calculate other ROC curve performance criteria such as the Youden index and misclassification cost, plot the ROC curve and other diagnostic measures. SAS also has a number of functions for ROC analysis, including PROC ROC: This method can be used to generate ROC curves, calculate the AUC, and compare the AUCs of two ROC curves. PROC LOGISTIC: This method can be used to fit logistic regression models and then to create ROC curves. PROC NLMIXED: This method can be used to fit mixed non-linear models, which can then be used to create ROC curves. In contrast to commercial software packages, the program R is a free OSS that contains all functions for the analysis of ROC curves using packages such as ROCR, pROC and optimal cutpoints. Among the R packages, ROCR is one of the most comprehensive packages for ROC curve analysis and contains functions for calculating the AUC with CI. pROC can be used to compare the AUC with the pAUC of different methods and provides CI for Se, Sp, AUC, and pAUC. Similar to ROCR, pROC also offers some functions for determining the optimal cut-off value, which can be determined using the Youden index and the Euclidean index. Optimal cut-points is a sophisticated R package specifically designed to determine the optimal cut-off point value [6]. Although these R packages have a large number of functions, they require good programming knowledge of the R language. A web tool for R-based ROC curve analysis, which includes easy ROC and plotROC, is a web-based program that uses the R packages such as plyr, pROC, and optimal cut-points to perform ROC curve analysis and extends the functionality of several ROC packages in R so that researchers can perform ROC curve analysis through an easy-to-use interface without having to write R code [29, 30].

### An illustration of different methods of cut-point selection with clinical data

In a clinical study of diagnostic accuracy of biomarkers, 30 patients of IBD and 30 healthy individuals were recruited based on pathologic examination [31]. The target population was patients who were referred to the outpatient clinics for their check-up for diagnosis of IBD. It was similar that physicians need to discriminate between IBD and healthy individuals in real life. All suspected patients underwent colonoscopy for pathology examination as gold standard. Then, blood samples were taken for all subjects to measure three biomarkers blindly including C-reactive protein (CRP), erythrocyte sedimentation rate (ESR) and malondialdehyde (MDA), were collected from 30 patients with inflammatory bowel disease (IBD) and 30 healthy control The equal sample size of IBD patients and healthy subjects were taken in order to achieve a higher statistical power of testing diagnostic accuracy. This 50% prevalence of IBD patients in our dataset does not influence the sensitivity and specificity of diagnostic biomarkers and thus it is not distorted the cut-point selection because the criteria for cut-point selection for all methods based on the sensitivity and specificity not based on PPV and NPV.

In our analysis, we applied the nonparametric ROC analysis to derive the AUC of different biomarkers and their 95% confidence interval (CI) in predicting IBD. The diagnostic accuracy of each biomarker in predicting IBD and the optimal cut-off point were calculated with 5 different methods for each biomarker using NCSS software. In addition, R software was also used to draw the density plot. The Youden index, Product, Euclidian, and IU, and DOR methods were used to determine the optimal cut-off point.

## Results

**Fig. 3:**
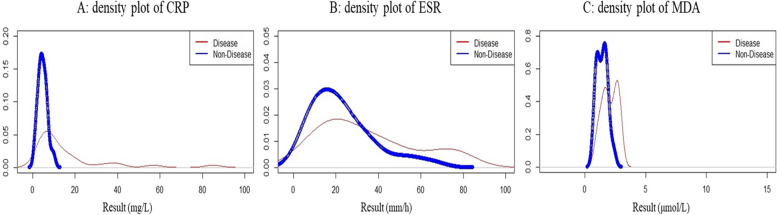
The density plot of the pairs of distributions of CRP, ESR, and MDA in the diseased (IBD) and the nondiseased individuals

Figure 3 displays the density plot of the pairs of distributions of three biomarkers including CRP, ESR, and MDA in IBD patients and healthy individuals. The distribution of CRP in healthy people was normal, but in IBD patients it had a large tail and extension on the right side and was skewed. ESR was elongated on the right side in both patients and healthy individuals. On description, the degree of elongation and skewness was greater in patients than in healthy individuals. The MDA value suggested a bimodal distribution in both patients and healthy subjects.

**Fig. 4:**
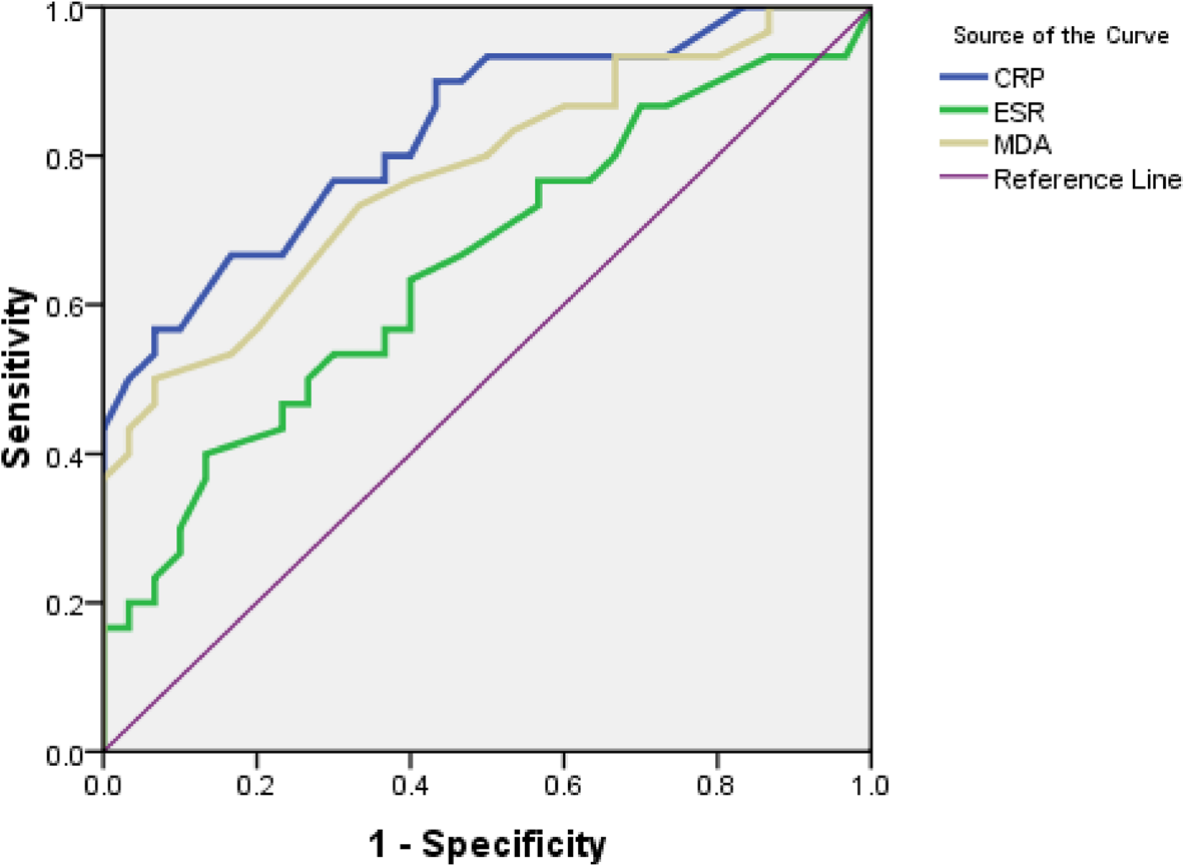
Nonparametric ROC curve of three Biomarkers of CRP, ESR and MDA in predicting IBD patients

Table 2 and Fig. 4 demonstrate the nonparametric ROC curves that all three biomarkers have significant predictive power, but CRP has a higher diagnostic accuracy than MDA and ESR. Table 3 indicates that the three Youden, Euclid, and Product methods have the same optimal cut-off point for CRP. As a result, Se and Sp were the same, and the IU estimated the cut-off point to be slightly below 6 mg/L. But the cut-off point of the DOR was at the upper extreme. Table 4 illustrates that the optimal cut-off point for the ESR is completely identical for the three Euclidian, Product, and IU methods, but differs significantly for the Youden method. The Youden method determined higher values (39 mm/h) for the ESR, which had a low Se. In contrast, the DOR method showed a limit value for the cut-off point. This obtained cut-off point had a high DOR, but compared to the Sp (Sp = 0.97), the Se (Se = 0.22) of this point was low. Table 5 represents that the optimal cut-off point for MAD is the same for the Euclidian, Product, and IU methods (1.7 μmol/L), but higher for the Youden method (2.1 μmol/L) with Se = 0.50 and Sp = 0.93. In contrast, the cut-off point of the DOR was higher (2.3 μmol/L), meaning that the DOR was maximal but had a low Se.

**Table 2:** The nonparametric AUC of different biomarkers in prediction of IBD and its 95%CI

Biomarkers	AUC	SE(AUC)	95% CI	*P*-Value
CRP	0.832	0.052	(0.731, 0.933)	0.001
ESR	0.653	0.071	(0.514, 0.792)	0.041
MDA	0.776	0.060	(0.658, 0.893)	0.001

**Table 3:** Result of cut-point selection with different methods by CRP and their diagnostic properties

Methods	Cut-point (mg/L)	Se (95%CI)	Sp (95%CI)	PPV (95%CI)	NPV (95%CI)	LR + (95%CI)	LR- (95%CI)	DOR (95%CI)
Youden	7	0.67 (0.47,0.82)	0.83 (0.65,0.94)	0.80 (0.59,0.93)	0.71 (0.53,0.85)	4 (1.17,13.58)	0.40 (0.12,1.36)	10 (2.94,33.95)
Euclidian	7	0.67 (0.47,0.82)	0.83 (0.65,0.94)	0.80 (0.59,0.93)	0.71 (0.53,0.85)	4 (1.17,13.58)	0.40 (0.12,1.36)	10 (2.94,33.95)
Product	7	0.67 (0.47,0.82)	0.83 (0.65,0.94)	0.80 (0.59,0.93)	0.71 (0.53,0.85)	4 (1.17,13.58)	0.40 (0.12,1.36)	10 (2.94,33.95)
DOR	10	0.50 (0.31, 0.68)	0.97 (0.82, 0.99)	0.94 (0.69, 0.99)	0.66 (0.50, 0.79)	15 (1.81,124.59)	0.52 (0.06,4.30)	29 (3.49,240.81)
IU	6	0.77 (0.57,0.90)	0.70 (0.51,0.85)	0.72 (0.53,0.86)	0.75 (0.55,0.89)	2.56 (0.77,8.55)	0.33 (0.90,1.09)	7.67 (2.95,32.82)

**Table 4:** Result of cut-point selection with different methods by ESR and their diagnostic properties

Methods	Cut-point (mm/h)	Se (95%CI)	Sp (95%CI)	PPV (95%CI)	NPV (95%CI)	LR + (95%CI)	LR- (95%CI)	DOR (95%CI)
Youden	39	0.40 (0.22,0.59)	0.87 (0.69,0.96)	0.75 (0.47,0.92)	0.59 (0.43,0.73)	3 (0.85,11.08)	0.69 (0.19,2.48)	4.33 (1.20,15.60)
Euclidian	22	0.63 (0.43,0.80)	0.60 (0.40,0.77)	0.61 (0.42,0.78)	0.62 (0.42,0.79)	1.58 (0.55,4.46)	0.61 (0.21,1.74)	2.59 (0.91,7.34)
Product	22	0.63 (0.43,0.80)	0.60 (0.40,0.77)	0.61 (0.42,0.78)	0.62 (0.42,0.79)	1.58 (0.55,4.46)	0.61 (0.21,1.74)	2.59 (0.91,7.34)
DOR	60	0.20 (0.07,0.38)	0.97 (0.82,0.99)	0.86 (0.42,0.99)	0.55 (0.40,0.68)	6 (0.74,59.27)	0.83 (0.09,7.33)	7.25 (0.81,64.45)
IU	22	0.63 (0.43,0.80)	0.60 (0,40,0.77)	0.61 (0.42,0.78)	0.62 (0.42,0.79)	1.58 (0.55,4.46)	0.61 (0.21,1.74)	2.59 (0.91,7.34)

**Table 5:** Result of cut point selection with different methods by MDA and their diagnostic properties

Methods	Cut-point (μmol/L)	Se (95%CI)	Sp (95%CI)	PPV (95%CI)	NPV (95%CI)	LR + (95%CI)	LR- (95%CI)	DOR (95%CI)
Youden	2.1	0.50 (0.31,0.68)	0.93 (0.77,0.99)	0.88 (0.63,0.98)	0.65 (0.49,0.78)	7.50 (1.43,35.49)	0.54 (0.10,2.67)	14 (2.81,69.56)
Euclidian	1.7	0.73 (0.54,0.87)	0.67 (0.47,0.82)	0.69 (0.49,0.83)	0.71 (0.51,0.86)	2.20 (0.72,6.70)	0.40 (0.13,1.22)	5.50 (1.81,16.68)
Product	1.7	0.73 (0.54,0.87)	0.67 (0.47,0.82)	0.69 (0.49,0.83)	0.71 (0.51,0.86)	2.20 (0.72,6.70)	0.40 (0.13,1.22)	5.50 (1.81,16.68)
DOR	2.3	0.43 (0.25,0.62)	0.97 (0.82,0.99)	0.93 (0.66,0.99)	0.63 (0.47,0.76)	13 (1.71,119.44)	0.58 (0.07,4.89)	22.18 (2.66,184.80)
IU	1.7	0.73 (0.54,0.87)	0.67 (0.47,0.82)	0.69 (0.49,0.83)	0.71 (0.51,0.86)	2.20 (0.72,6.70)	0.40 (0.13,1.22)	5.50 (1.81,16.68)

## Discussion

Defining the optimal cut-points for quantitative biomarkers plays a crucial role in clinical decision-making in diagnostic medicine. ROC analysis is an optional choice for determining the optimal cut-off value. However, there is no single standard method to determine the optional cut-off value of biomarkers. As illustrated in this comprehensive review, several methods have been proposed in the context of ROC analysis. The best known is the Youden index due to its clinical interpretation, which maximizes the proportion of correct classification after correcting for the random level. In some scenarios of the underlying distributions of biomarkers, especially for binormal distributions with equal variance, the Euclidean index, which maximizes the points on the ROC curve from the left corner of the ROC space at (0,1), may be more accurate than the Youden index [9], but these two methods gave a similar estimate of the cut-point in the ROC space in the above scenario [15].

Our findings in clinical investigation of biomarkers in IBD patients showed that the density function of ESR and CRP was skewed to the right tail, but not the distribution of CRP in healthy individuals. While the density function of MDA indicated a bimodal shape in both IBD patients and healthy individuals. Despite the presence of bimodal shapes and a right-skewed distribution, the three Euclidean, Product and IU metrics yielded quite similar estimates of the optimal cut-off points, but the Youden index yielded a higher cut-off value. The greatest inconsistency was found in DOR compared to other metrics. It always yielded the optimal cut-point in the critical tail. Our findings are in accordance with the results of other studies [15, 32]. The inconsistency of the results of DOR is related to the convex distribution of log(DOR) as a ratio metric. In particular, for a pair of Gaussian distributions, the metric of log(DOR) is U-shaped across different cut-points [15, 32].

In several studies, population-based biomarker distributions and Monte Carlo simulation studies with repeated samples have shown that the three Youden, Euclidean and Product methods yield similar estimates of cut-points under certain conditions of Gaussian distributions [15]. However, log(DOR) results in a higher/extreme value of the cut-point, which has very low validity and reliability. Hajian–Tilaki investigated the population distribution based on test results and suggested in some scenarios of the data from the bilogistic model in diseased and non-diseased individuals that log(DOR) itself is noninformative and its metric is flat across the value of the different cut-points [15].

For the clinical practice of determining cut-points, sample data were used in the current study to illustrate the practical application of the NCSS software in cut-point selection. Software has been developed for cut-point selection in clinical research as described in this detailed review. The SPSS software does not offer this calculation directly. The R software in the ROC analysis library does offer these optimal cut-points, but may be more specialized and less familiar to clinicians. In our experience, a practitioner can use the NCSS software to create an estimate of the optimal cut-points using at least five methods in the ROC analysis: Youden index, Euclidean index, Product method, IU and DOR.

The present study provided a practical example and indicated how the optimal cut-points can be calculated in clinical research. We have shown that in some scenarios, the four common methods for selecting optional cut-points can lead to identical results. However, the inconsistency of cut-point selection is possible in some other conditions of test results with skew distributions or bimodal form.

The results of the ongoing study on the clinical example of biomarker data for prediction of IBD represented that the four Youden, Euclidean, Product of Se and Sp and IU methods gave a similar cut-point for CRP, but DOR gave a higher value for cut-point selection. Nevertheless, for ESR and MAD, the Youden index gave different results than Euclidean, Product and IU methods. This inconsistency may depend in part on the underlying distributions of test scores in diseased and healthy populations that we have shown the density function of test results with graphical presentation. The higher degree of skewness and heterogeneous variance may lead to greater inconsistency in the results. In our example, the extreme value of the cut-point of DOR can be explained by the convex distribution of log(DOR) as a ratio criterion. This result is consistent with other findings in the selection of cut-points [32]. Thus, extensive Monte Carlo simulation studies are needed to explore the conditions for the distribution of test results that may lead to inconsistent results by different methods for the cut-point in the evaluation of clinical diagnostic tests. We had a small sample dataset and all data was used for training model. Thus, our study may limit to lack of external dataset for cross-validation of diagnostic performance of calculated optimal cut-points with different methods because the diagnostic performance of selected cut-points was calculated with training dataset only.

## Conclusion

Overall, the four methods including Youden index, Euclidean index, Product, and IU can produce quite similar optimal cut-points for binormal pairs with the same variance. The cut-point determined with the Youden index may not agree with the other three methods in the case of skewed distributions while DOR may not produce valid informative cut-points. Therefore, more extensive Monte Carlo simulation studies are needed to investigate the conditions of test result distributions that may lead to inconsistent results in clinical diagnostics.

## Data Availability

Data cannot be shared openly but are available on request from corresponding author.

## Abbreviations

ROC: Receiver operator characteristics
D: Diseased
ND: Nondiseased
Se: Sensitivity
Sp: Specificity
NPV: Negative predictive value
PPV: Positive predictive value
TPF: True positive fraction
TNF: True negative fraction
FNF: False negative fraction
FPF: False positive fraction
AUC: Area under the curve
IU: Index of union
DOR: Diagnostic odds ratio
C: Cost
LR +: Positive likelihood ratio
LR-: Negative likelihood ratio
NNM: Number needed to mis diagnosed
pAUC: Partial area
IBD: Inflammatory bowel disease
CRP: C-reactive protein
MDA: Malondialdehyde
ESR: Erythrocyte sedimentation
